# Frequency‐dependent deficits in head steadiness in patients with nonspecific neck pain

**DOI:** 10.14814/phy2.14013

**Published:** 2019-03-04

**Authors:** Ann‐Katrin Stensdotter, Ingebrigt Meisingset, Morten Dinhoff Pedersen, Ottar Vasseljen, Øyvind Stavdahl

**Affiliations:** ^1^ Department of Neuromedicine and Movement Science The Norwegian University of Science and Technology NTNU Trondheim Norway; ^2^ Department of Public Health and Nursing Faculty of Medicine and Health Sciences The Norwegian University of Science and Technology NTNU Trondheim Norway; ^3^ Department of Engineering Cybernetics Faculty of Information Technology and Electrical Engineering The Norwegian University of Science and Technology NTNU Trondheim Norway

**Keywords:** Frequency responses, reflex mechanisms, stability, stiffness, voluntary control

## Abstract

Motor control impairments are reported in patients with nonspecific neck pain but the particular deficits in underlying regulatory systems are not known. Head steadiness is controlled both by voluntary and reflex systems that are predominantly effective within different frequency intervals. The aim of the present study was to investigate within which frequency range(s) potential motor control deficits may reside. The ability to keep the head stationary in space in response to unpredictable perturbations was tested in 71 patients with nonspecific neck pain and 17 healthy controls. Participants were exposed to pseudorandom horizontal rotations across 10 superimposed frequencies (0.185–4.115 Hz) by means of an actuated chair in three conditions; with a visual reference, and without vision with, and without a cognitive task. Below 1 Hz, patients kept the head less stable in space compared to healthy controls. Between 1 and 2 Hz, the head was stabilized on the trunk in both groups. Patients kept the head more stable relative to the trunk than relative to space compared to healthy controls. This was interpreted as higher general neck muscle co‐activation in patients, which may be explained by altered voluntary control, or/and upregulated gamma motor neuron activity which increases the contribution of reflex‐mediated muscle activation. Alternatively, increased muscle activity is secondary to vestibular deficits.

## Introduction

Impaired control of the head and neck in nonspecific neck pain is reported in several studies showing delayed onset and reduced activity in neck muscles (Falla 2004), deficits in direction specific force production and increased muscle co‐activation (Lindstrom et al. 2011), reduced freedom of movement (Woodhouse and Vasseljen 2008), increased general stiffness and rigidity of movement (Meisingset et al. 2015), and jerky and irregular cervical movements (Sjolander et al. 2008). Studies have in general assessed voluntary neck movements (Sjolander et al. 2008), such as tracing an outlined figure (Woodhouse 2010) or tracking an unpredictably moving target (Kristjansson et al. 2004). Other tests have assessed the ability to keep the head steady against the force of gravity (Woodhouse et al. 2010; Meisingset et al. 2015). Several of the tests used are shown to measure different aspects of sensorimotor control (de Zoete et al. 2018). Notably, these voluntary tasks also allow to a large extent individual strategy which increases variability within and between persons, thereby limiting the probability to uncover and define specific neurophysiological impairments.

Correlations between fear of movement and neck kinematics (Sarig Bahat et al. 2013) suggest that impaired control may reside within the voluntary domain of regulation of muscle stiffness, whereas similar impairments demonstrated in whiplash‐associated neck pain have been ascribed to possible deficits in reflex mechanisms (Treleaven et al. 2011). Studies are however typically dedicated to describing impaired control on a performance level and protocols are seldom designed to investigate deficits in underlying neurophysiological motor control systems. To tease out underlying mechanisms, tests that eliminate individual strategy are needed to assess sensorimotor control subsystems.

Two reflex systems are proposed to contribute to motor control of the head and neck by regulating muscle stiffness; the vestibulocollic reflex (VCR) keeps the head stable in space by vestibular neurons projecting to neck motor neurons, activating neck muscles to produce compensatory head movements in the opposite direction and inhibiting muscles producing forces in the same direction relative to the perturbing force (Wilson and Schor 1999). The cervicocollic reflex (CCR) keeps the head and neck stable relative to the trunk by means of proprioceptive input from muscle spindles (Peterson 2004), activating muscles working with the direction of the perturbation when those are exposed to stretch. During voluntary movements, reflex activity has to be canceled for the head to move freely (Roy and Cullen 2001, 2004). Alternatively, voluntary activity may modulate reflex excitability where reflexes serve to dampen the oscillations created by the mass‐spring system of the head and neck (Peng et al. 1996) to provide a basic level of muscle stiffness. Although theoretical assumptions about reflex control of the head and neck still remain to be directly demonstrated experimentally (Goldberg and Cullen 2011), indirect methods may be applied. By studying motor responses to controlled perturbations, it may be possible to infer whether impaired control of the head and neck in patients with nonspecific neck pain may be dominated by changes in reflex or voluntary regulation of muscle stiffness. Knowledge of the underlying mechanisms of impaired control is imperative to develop more effective and targeted treatment to normalize deficits observed in patients with neck pain.

This project used a protocol originally designed by Keshner and Peterson (1995), assessing head stability in space during exposure to horizontal plane pseudorandom rotations of different and superimposed frequencies. Below 1 Hz, fair compensation to perturbations suggests that voluntary control is predominant. Between 1 and 2 Hz, unity between the head and trunk movements indicates that reflexes stabilize the head relative to the trunk. Above 2 Hz, resonance emerges meaning that the head moves more than the trunk (Keshner and Peterson 1995; Keshner et al. 1995; Peng et al. 1996, 1999). This protocol could however not discriminate between the two reflex systems, VCR and CCR. The present study repeated the same protocol used by Keshner and Peterson (Keshner and Peterson 1995) to study control of the head in space in the different frequency ranges.

The aim of the present study was to investigate within which frequency range(s) potential motor control deficits may reside. Specifically, whether potential changes underlying motor control impairments in patients with nonspecific neck pain reside in the frequency range controlled by reflex systems or in the frequency range predominantly under voluntary control, or both. The ability to keep the head stable in space during perturbations to the body of different frequencies was explored.

## Methods

### Participants

Patients referred for treatment were recruited from community and hospital physiotherapy clinics (*n* = 71). This study was part of a larger study (Meisingset et al. 2015) and all patients who completed the presented protocol were included. Inclusion criteria were nonspecific neck pain without radiation below the elbow, pain duration >2 weeks, and pain intensity ≥3 on a numerical rating scale (NRS, no pain – worst pain, 0–10) on the day of testing. The control group consisted of staff and students (*n* = 17) without neck and shoulder complaints. The number of control persons to establish normality was based on previous studies (Keshner and Peterson 1995). Exclusion criteria were reduced and uncorrected vision or diagnosed vestibular deficits, orthopedic or neurological conditions (Table 1). The study was approved by the Regional Ethics Committee (2011/2522/REK) and conducted in agreement with the Helsinki declaration. Participants signed an informed consent before entering the study.

**Table 1:** Subject characteristics

Variables	Neck pain *n* = 71	Healthy controls *n* = 17
Gender, female (*n* [%])	50 (70)	9 (53)
Age	44.0 (12.9)	31.5 (7.4)***
Body mass index	24.2 (3.7)	23.7 (2.9)
Current neck pain intensity (NRS; 0–10)	4.7 (1.4)	–
Worst neck pain last month (NRS; 0–10)	7.3 (1.5)	–
Duration of neck pain >3 months (*n* [%])	60 (90)	–
Number of pain sites (*n* [%])		
Only neck pain	14 (20)	
≥2 additional pain sites	34 (49)	
Neck disability index (NDI; 0–100)	31.7 (12.2)	–
Patient‐specific functional scale (PSFS; 0–10)	6.5 (2.0)	
Tampa scale of kinesiophobia (TSK; 13–52)	24.7 (4.2)	
Pain catastrophizing scale (PCS; 0–52)	12.4 (7.8)	
Pain self‐efficacy scale (PSES; 0–60)	44.8 (10.1)	
Self‐rated general health (*n* [%])		
Fair	30 (43)	
Good	38 (54)	
Very good	2 (3)	
Use of analgesic (*n* [%]	33 (48)	

Given values are mean (SD), unless otherwise stated.

NRS = numerical ratings scale.

*** *P* < 0.001.

### Data acquisition

Head steadiness in space was assessed while the body was exposed to pseudorandom rotations in the horizontal plane. Each participant was exposed to one trial (duration 200 sec) of each of three conditions, all in the same and following order in accordance with the original protocol by Keshner and Peterson (1995); with vision (VS), without vision (NV), and without vision with an additional mental task counting backwards from 500 in steps of seven (MA), the latter in order to divert attention from conscious control of head position. VS aimed to investigate voluntary control with a visual reference provided by a laser pointer mounted in a rigid fixture on the head aimed toward a vertical line on a white surface 1.6 m in front of the participant. A 5 cm intersecting horizontal line guided the projected laser beam in order to keep the head stable in neutral position by keeping the laser beam aligned in the horizontal plane. NV challenged voluntary control without visual information. The purpose of MA was to investigate the contribution of reflex control.

**Figure 1:**
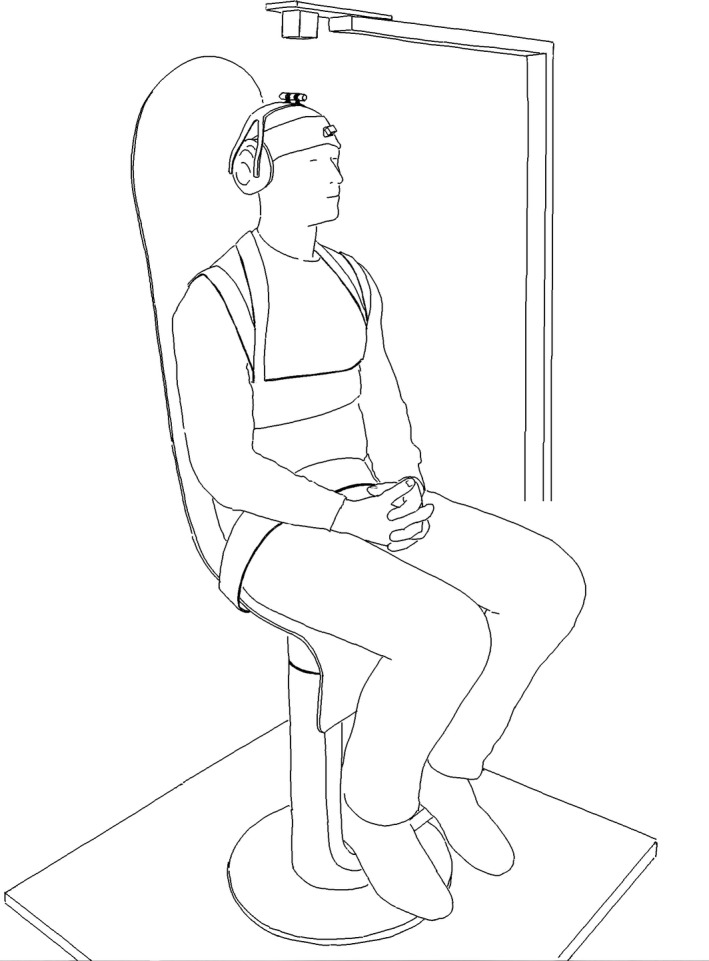
An instrumented participant strapped to the actuated chair. The cube above the persons’ head represents the electromagnetic transmitter. Note that the ear muffs have holes leaving the ears uncovered and hearing intact (Stensdotter et al., Physiological Reports, 2016).

Sinusoidal rotations around the vertical axis were induced to the trunk by means of an actuated chair, the rotational axis coinciding approximately with the axis of the cervical spine. The participant was seated firmly strapped to the backrest and seat to minimize movement between the body and the chair (Fig. 1). Only the head was allowed free movement. Cross correlations from pilot studies assured that the frequency responses of the trunk corresponded to those induced by the chair *φ*_*xy*_ (*τ* = 0) = 0.95. The rotation was driven by a brushed DC‐motor with a 1:308 gear ratio (Maxon Motor, Sachseln, Switzerland, part no. 353295), controlled by a LabVIEW program via a NI 9505 DC Brushed Servo Drive (both of National Instruments Corporation, Austin, TX). To minimize the influence on the electromagnetic motion capture system, the main structure of the chair was built from nonmetallic materials. The DC‐motor and gear were placed close to the floor and power electronics were placed 2 m away from the base of the chair. Data for rotations in the horizontal plane were registered by three sensors placed on the chair, on the back of the participant at the level of the second thoracic vertebrae, and on the forehead, and collected at 240 Hz by a Liberty electromagnetic motion tracking system (Polhemus, Colchester, VT). The transmitter was placed ~20 cm above the head of the subject, and all sensors stayed within a 50 cm distance from the transmitter throughout the experiment (Fig. 1).

The construction of the sum‐of‐sines excitation signal was based on the original function provided by Keshner and Peterson (Keshner and Peterson 1995) and consisted of 10 superimposed harmonic components chosen as prime multiples of a base frequency *F *=* *0.005 Hz. The prime numbers used for the harmonic multiples were:

$$H=\left\{37,49,71,101,143,211,295,419,589,823\right\}$$

The sinusoids of relative primes provided pseudorandom perturbations in a pattern without repetitions over the fundamental period of *T* = 200 sec, preventing anticipatory preparation in the participants and contamination between the resulting frequencies (0.185–4.117 Hz). Chair velocity amplitudes were decreased as frequency increased: 20°/sec from 0.185 to 0.355 Hz, 19°/sec from 0.505 to 1.055 Hz, 16°/sec from 1.475 to 2.095 Hz, 15°/sec at 2.945 Hz, and 13°/sec at 4.115 Hz. The maximum rotational excursion occurred at the lowest frequency and was approximately ±17°. The same waveform was used for all conditions and all participants. The sum‐of‐sines angular velocity excitation signal, denoted by *u*(*t*), may be described by the function

$$\begin{array}{c} u\left(t\right)=\sum_{k\in H}a_{k}\sin\left(2\pi Fkt+\phi_{k}\right), \end{array}$$

where *k* represents each of the harmonics, *a*_*k*_ is the amplitude of the *k*′th harmonic (in radians/second), *t* is time (in seconds), and *ϕ*_*k*_ is the phase angle (radians) of the *k*′th harmonic at *t* = 0. In the current case, *ϕ*_*k*_ = 0∀*k*. This excitation induced sinusoidal rotations in the trunk and head, given approximately by the formulae

$$\theta^{T}\left(t\right)\approx \sum_{k\in H}A_{k}^{T}\sin\left(2\pi Fkt+\phi_{k}^{T}\right)$$

$$\theta^{H}\left(t\right)\approx \sum_{k\in H}A_{k}^{H}\sin\left(2\pi Fkt+\phi_{k}^{H}\right)$$

The superscripts signify whether the trunk (*T*) or head (*H*) angle is in question. To signify that these angles are both measured with respect to a room‐fixed coordinate frame, the quantities *θ* ^*T*^ and *θ* ^*H*^ will be referred to as the trunk‐room angle and the head‐room angle, respectively. Similarly, the angle of the head with respect to the trunk will be referred to as the head‐trunk angle. Note that these measured signals will include frequency content (noise) not present in the excitation signal, implying that the above formulations are not exact, hence the approximation signs.

Also, note that the excitation signal is defined in terms of the angular velocity, $u\left(t\right)$, while *θ* ^*T*^(*t*) and *θ* ^*H*^(*t*) are angles. The above formulae still hold as the angular excursion amplitude coefficients [*A*_*k*_] relate to the angular velocity amplitude coefficients as

$$A_{k}=-\frac{1}{2\pi Fk}a_{k},k\in H.$$

### Data analysis

**Figure 2:**
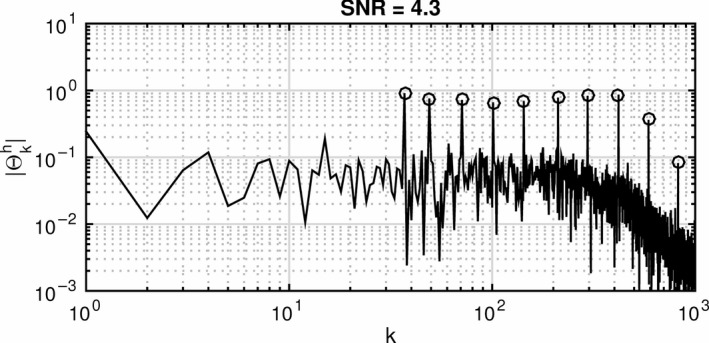
Spectral magnitudes of head movements in response to rotational perturbations in the horizontal plane, showing signal‐to‐noise ratio (SNR). The *y*‐axis shows the amplitudes and the *x*‐axis the harmonics. Excitation harmonics are indicated by circles. The figure displays that even the smallest rotations at 4.115 Hz were detected by the system. No significant over‐harmonics were detected which allows the assumption of linearity for analysis of the system (Stensdotter et al., Physiological Reports, 2016).

Information about the participants’ motor responses to the perturbation was extracted with spectral analysis. Under the assumption of linearity, the motion of the head and trunk would be a sum‐of‐sines in the excitation frequencies. This assumption was validated with analyses of the spectral magnitudes for the head‐room and trunk‐room angles, showing satisfactory signal‐to‐noise ratio (SNR) and without notable over‐harmonic content, albeit with quite notable noise levels for the head‐room angle, particularly at the highest frequencies (Fig. 2). The excitation response of a linear dynamic system may be modeled as a complex transfer function, which describes the gain and phase shift of the output (response) signal relative to the input (excitation) signal. Such a function may be expressed as

$$G\left(j2\pi f\right)=\frac{Y(j2\pi f)}{U(j2\pi f)},\left(\frac{\mathrm{Response}}{\mathrm{Excitation}}\right),$$

where *Y* and *U* are polynomial functions, *j* is the imaginary unit and *f* is the frequency in Hz. The transfer function relating the head‐room angle (response) to the trunk‐room angle (excitation) was estimated by calculating a Fourier series of the recorded time series over the excitation frequencies. Computation of the following integrals furnished a complex signal description at the *k*′th harmonic:

$$\Theta_{k}^{T}=\frac{2}{T}\int_{0}^{T}\theta^{T}\left(t\right)e^{-j2\pi Fkt}\;dt,\;\Theta_{k}^{H}=\frac{2}{T}\int_{0}^{T}\theta^{H}\left(t\right)e^{-j2\pi Fkt}dt$$

The transfer function was subsequently evaluated at the discrete excitation frequencies by evaluating

$$G_{k}=\Theta_{k}^{H}/\Theta_{k}^{T},k\in H$$

Gain and phase shifts of the head‐room angle relative to the trunk‐room angle were recovered by taking the absolute value and argument (angle) of this complex transfer function, as follows:

$$\frac{A_{k}^{H}}{A_{k}^{T}}=\left|G_{k}\right|,\phi_{k}^{H}-\phi_{k}^{T}=\arg G_{k}$$

Resulting transfer functions are presented as Bode plots with gain and phase shown for the 10 excitation frequencies. The Bode plot decouples the system properties of gain, phase shift and time constants/Eigen frequencies, which allows direct comparison and statistical analyses of linear systems with different dynamics, expected in human bodies of different size and mass. Thus, our statistical analysis is also based on Bode data (i.e., decimal logarithmic gain and linear phase).

In order to enable comparison with previous publications (Keshner and Peterson 1995; Stensdotter et al. 2016), Figure 3 shows gain on a logarithmic scale on the left axis, while the right axis shows the decimal logarithm of this factor plotted on a corresponding linear scale. In the following, we present both quantities; the case of unity gain, for example, will be presented as “gain = 1 (log10(1) = 0)”, where “1” and “0” relate to the left and right axes, respectively. Statistics are based on the decimal logarithm (right axis). Theoretically, perfect compensation for the head in response to the perturbations would be represented by a gain of zero (left axis), that is, the head is kept stationary in space and thus has no angular amplitude relative to the room at the excitation frequency in question. This is achieved by head‐trunk rotations of the same amplitude as, but in the opposite direction of, that of the trunk‐room angle. Gain = 1 (unity, left axis) indicates that the head moves in space with the same amplitude as the trunk, and gain >1 (left axis) indicates that the head moves more than the trunk relative to space. Perfect temporal compensation in response to perturbations would be shown as 0° shift for phase angles; positive values denote phase lead and negative values indicate phase lag.

**Figure 3:**
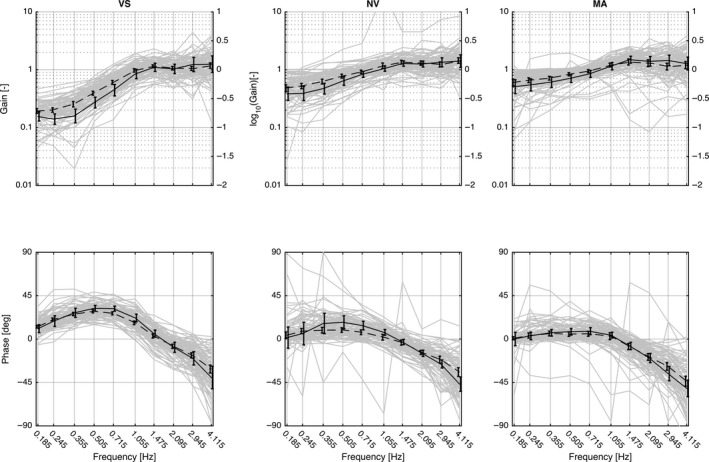
Bode diagrams of transfer functions for the three conditions with vision (VS), without vision (NV), and without vision with a cognitive task (MA), respectively. Mean and 95% CI. Plotted values are not adjusted for age and gender. Solid line: healthy controls, dashed line: patients. Gray curves in the background show the individual responses. Statistics are based on decimal logarithms shown on the right axis.

### Statistical analysis

Kinematic rotational data generally need to be treated with special statistical methods, for example, the Cosine statistics (Stavdahl et al. 2005), that account for the inherent cyclicity of rotations. However, for comparison of phase shifts of different transfer functions, traditional statistical methods were employed in order to avoid for example, treating two phase angles as the same if they differ by a multiple of 2π radians. The statistics were generated with SPSS 22.0 (Statistical Package for the Social Sciences, Inc., Chicago, ш). Normal distribution was confirmed with Q‐Q and P‐P plots. For gain and phase, separate general linear models were constructed for repeated measures with conditions as within subject factors (*n* = 3, VS, NV, MA) with frequencies (*n* = 10) as measures within each condition. Differences between groups (*n* = 2: neck pain and control) were assessed with multivariate analysis (Wilk's Lambda) across conditions with Bonferroni corrections for multiple comparisons. Sphericity was assumed according to Mauchly's test. Post hoc linear regressions were used to assess group differences for separate measures within each condition. Due to differences between the groups, age and gender were used as covariates in all analyses. Alpha‐level: *P* < 0.05.

## Results

### Gain

The perturbations produced similar general patterns across groups and conditions with increasing gains as an effect of higher frequencies, however with significant effects of group and of condition. Multivariate analyses showed a significant effect of group for at least one measure across conditions (*F*_10,72_: 2.6, *P* = 0.009) and a significant effect of condition (*F*_20,304_: 27.4, *P* < 0.001). Between 0.185 and 1.055 Hz, the patient group displayed higher mean gain than the control group in the VS condition. At frequencies above 1.055 Hz, no group differences were found. In the NV condition, the patient group displayed higher gain than the control group between 0.245 and 0.715 Hz. In the MA condition, higher gain was found in the patient group only at 0.505 Hz (Table 2, Fig. 3).

**Table 2:** Comparisons between patients with neck pain and healthy controls for each separate condition (with and without vision, and without vision with a cognitive task)

Frequency (Hz)	Group mean difference (95% CI)		
	VS	NV	MA
Gain			
0.185	**0.13 (0.02, 0.23)** *	0.11 (−0.04, 0.26)	0.03 (−0.12, 0.17)
0.245	**0.17 (0.067, 0.28** **	**0.16 (0.03, 0.29)** *	0.07 (−0.04, 0.18)
0.355	**0.21 (0.08, 0.34)** **	**0.16 (0.05, 0.27)** **	0.07 (.−0.02, 0.16)
0.505	**0.18 (0.09, 0.28)** ***	**0.12 (0.03, 0.21)** **	**0.09 (0.02, 0.16)** *
0.715	**0.17 (0.07, 0.26)** **	**0.08 (0.01, 0.15)** *	0.05 (−0.02, 0.13)
1.055	**0.10 (0.21, 0.18** *	0.10 (−0.01, 0.21)	0.02 (−0.06, 0.11)
1.475	0.05 (−0.03, 0.12)	0.06 (−0.02, 0.14)	−0.06 (−0.19, 0.07)
2.095	0.04 (−0.05, 0.12)	0.05 (−0.04, 0.14)	−0.04 (−0.19, 0.10)
2.945	−0.03 (−0.13, 0.08)	−0.00 (−0.11, 0.11)	−0.08 (−0.23, 0.06)
4.115	−0.03 (−0.17, 0.10)	0.05 (−0.08, 0.17)	−0.03 (−0.19, 0.12)
Phase			
0.185	2.1 (−5.6, 9.7)	−4.6 (−24.0, 14.8)	8.1 (−3.1, 19.3)
0.245	1.6 (−8.0, 11.1)	4.8 (−13.5, 23.0)	3.8 (−9.3, 17.0)
0.355	1.3 (−8.2, 10.9)	−0.503 (−21.6, 20.6)	−1.2 (13.4, 11.0)
0.505	−5.3 (−13.4, 2.8)	3.6 (−18.5, 25.8)	−7.0 (−18.5, 4.5)
0.715	−**11.2 (**−**19.2,** −**3.1)** **	7.7 (−16.6, 32.1)	−10.8 (−23.2, 1.6)
1.055	−**13.5 (**−**23.1,** −**3.8)** **	11.8 (−15.4, 39.0)	−12.8 (−29.9, 4.3)
1.475	−**12.3 (**−**22.6,** −**1.9)** *	9.7 (−20.7, 40.1)	−14.2 (−34.1, 5.6)
2.095	−5.4 (−16.0, 5.2)	11.8 (−21.6, 45.3)	−8.1 (−29.1, 12.9)
2.945	−8.8 (−22.8, 5.1)	14.1 (−19.4, 47.5)	−1.0 (−26.9, 24.9)
4.115	2.5 (−19.1, 24.1)	28.5 (−7.1, 64.1)	10.9 (−19.1, 40.8)

Estimated group difference with 95% confidence intervals (CI) within each separate condition adjusted for age and gender. Positive values indicate higher gain and phase for patients compared to healthy controls. Estimates for gain correspond to decimal logarithms shown on the right axis in Figure 3, while estimates for phase angles are linear.

VS, with vision; NV, without vision; MA, without vision + cognitive task.

Level of significance: *P < 0.050, **P < 0.010, ***P < 0.001 are in bold.

### Phase

Akin to gain, the perturbations produced similar general patterns across groups and conditions. A phase lead (>0°) was seen for lower frequencies, while an increasing phase lag (<0°) was seen with higher frequencies. Multivariate tests showed no significant effect of group across conditions (*F*_10,72_: 1,3, *P* = 0.265); however, 95% CI in the graphs indicated some localized groups differences. Post hoc tests were therefore performed for separate conditions (VS, NV and MA) showing significantly greater phase lead in the control group in VS at 0.715 and 1.055 Hz (Table 2, Fig. 3). Within subjects’ multivariate tests showed a significant effect of condition (*F*_20,304_: 10.2, *P* < 0.001).

## Discussion

### General motor responses

Our results lend further support to previous findings of impaired motor control of the head and neck in nonspecific neck pain (Falla et al. 2004; Kristjansson et al. 2004; Sjolander et al. 2008; Woodhouse and Vasseljen 2008; Woodhouse et al. 2010; Meisingset et al. 2015), suggesting increased stiffness of the system as a possible common denominator contributing to explain motor control difficulties. Patients showed a general pattern of keeping the head steadier relative to the trunk rather than in space when exposed to perturbations to the body in the horizontal plane. This response was particularly evident in the frequency range where compensation to perturbations can be controlled by voluntary activity. Compensation in this range became however successively worse when vision was removed and when in addition attention was diverted.

### Measurement system limitations

The Polhemus (Polhemus Liberty Specifications, 2017) range of motion tracking instruments employ AC magnetic fields that are measured sequentially in order to determine each sensor's position and orientation relative to the transmitter. Sensor movement during this measurement sequence inevitably induces what can be denoted dynamic measurement errors. Several groups, for example, Hassan et al. (2007) and Nafis et al. (2006), have quantified various aspects of dynamic accuracy and precision of electromagnetic trackers, but due to the use of different motion tracker technology and/or different experimental conditions, their results cannot readily be applied or extrapolated to our study. For this reason, our results related to the higher excitation frequencies, small angular excursion, and intersubject variability (i.e. >3 Hz) may be regarded as uncertain. For the lower frequency range, however, we expect the effective measurement error to be comparable to the static RMS error of 0.15° stated by the manufacturer. Limitations in static accuracy will only influence the analysis at zero frequency, which is not of interest in this study. Furthermore, random errors induced by limited instrument precision will be smoothed out during the Fourier analysis. In light of all this, we propose that our results based on the lower frequency band up to 2 Hz are valid both qualitatively and quantitatively.

### Methodological considerations

Some outliers are seen in the dataset across frequencies as well as across groups, indicating that not all subjects were able to perform the task. As not being able to perform the task could potentially have been the distinction between patients and controls, those were not removed from the dataset. Statistics with or without these outliers did however not change the results. There were no correlations between outcome variables for gain or phase with background variables for participants’ characteristics (Table 1), explaining variability or outliers in the dataset. As the task becomes increasingly difficult when moving from condition VS through NV to MA, it should be expected that the participants’ compensation would lag increasingly more behind the external perturbation as frequency increases. However, some positive phase shifts were also evident with increasing frequencies. In order to test whether these were artifacts, different conventions were tried with effect only on one single outlier. Note that there are no absolute criteria for choosing one convention over the other. In the present study, the Matlab function “*P* = angle (*Z*)” was used which returns the phase angles, in radians, for each element of complex array *Z*. The angles lie between ±*π*. Furthermore, with regard to the dynamic properties of compensatory head movements generated by voluntary, reflex, and mechanical mechanisms, total compensation, for example, zero gain and 0° phase angle are not attainable. The voluntary system is likely to produce a compensatory signal with phase opposite to that of the imposed rotation of the trunk with a delay of approximately 0.2 sec. (Keshner and Peterson 1995). Positive phase shift has also been observed in other studies (Baker et al. 1985; Goldberg and Peterson 1986; Keshner and Peterson 1995; Forbes et al. 2013; Stensdotter et al. 2016). It is suggested that VCR would contribute to dampen the system dynamics, potentially contributing to the phase lead (Peng et al. 1996; Forbes et al. 2013). Notably, phase lead was significantly smaller in patients than control between 0.715 and 1.055 Hz.

Assumptions about linearity were made similar to previous studies (Keshner and Peterson 1995; Forbes et al. 2013). When the nature of nonlinearity is unknown, linearity is assumed as the most robust method.

### Responses below 1 Hz

The most apparent difference between groups was found in the condition with access to visual reference (VS) and at frequencies equal to or below 1 Hz, where head steadiness in space is maintained predominantly under voluntary control (Guitton et al. 1986; Keshner and Peterson 1995). Therefore, the present findings may be interpreted as depending on deficits in voluntary control in patients, causing increased stiffness and reduced ability to compensate for perturbations in order to keep the head steady in space. Note that voluntary control is presumed predominant and does not exclude the involvement of reflex mechanisms. It has been debated whether reflex control contributes to dampen oscillations of the mass‐spring system of the head and neck (Peng et al. 1996, 1999) or if voluntary activity completely can override reflex responses (Roy and Cullen 2001, 2004). In a recent study on anteroposterior perturbations between 0.3 and 8 Hz, the estimated contributions of VCR and CCR were fitted to responses of healthy persons, showing that reflexes are present also at low frequency perturbations. This study did however not include aspects of voluntary control (Happee et al. 2017).

Both alternatives describe that head steadiness in space can be controlled voluntarily at frequencies below 1 Hz, but do not refute the possible involvement of altered reflex control as an explanation to increased stiffness. Notably, in the MA condition when attention was directed toward a cognitive task, the movement of the head nearly followed that of the trunk in both groups with similar pattern as in the VS and NV conditions. This leaves two interpretations of the motor responses in patients: (1) neck muscle stiffness is enhanced by voluntary activation, or (2) reflex responses are enhanced increasing neck muscle stiffness. Both alternatives may potentially explain greater head steadiness relative to the trunk in patients in conditions (VS and NV) where attention was turned toward conscious control of head position. In MA, however, when attention was diverted from conscious control of head position, reflexes appear, at least to some extent, to stabilize the head relative to the trunk also at low frequencies (Guitton et al. 1986; Keshner and Peterson 1995). This notion supports alternative two, which suggests that reflexes are active also at lower frequency perturbations. Notably, in MA, patients displayed gain closer to one at 0.505 Hz. Further support for alternative two is found in the hypothesis by Johansson and Sojka (1991), suggesting a vicious circle where increased muscle tension produces metabolites activating gamma motor neurons, increasing activity in muscle spindle afferents again increasing muscle stiffness (Johansson and Sojka 1991). Pain induced increase in gamma motor neuron activity has been corroborated in studies of experimental muscle pain showing increased amplitude of the stretch reflex but without a corresponding increase in the H‐reflex amplitude (Matre et al. 1998). Reflex‐mediated increase of muscle stiffness would restrict freedom of movement for the head and neck, a freedom which is necessary to voluntarily compensate for perturbations to the trunk when attempting to keep the head steady in space. This reasoning is corroborated by other studies on neck pain (Boudreau and Falla 2014; Cheng et al. 2014), suggesting increased co‐activation between agonist and antagonist muscles of the neck in response to unpredictable perturbations. Alternatively, increased stiffness observed in patients may be secondary to vestibular deficits. Cervical afferents are involved in the cervico‐ocular reflex (COR) which operates in conjunction with the vestibulo‐ocular reflex (VOR). Increased COR has been reported in nonspecific neck pain (de Vries et al. 2016). In elderly, increased COR seem to compensate for reduction of sensory loss in the vestibular system (Kelders et al. 2003). As the COR operates in conjunction with VOR and is elicited by proprioceptive input from facets joints and deep muscles of the neck, increased stiffness of the neck may compensate for visual acuity (Crane and Demer 1998) where stability on the retina is reduced due to vestibular deficits.

### Responses between 1 and 2 Hz

In the frequency range between 1 and 2 Hz, unity between head and trunk movement occurs, that is, the head moves with the trunk (Keshner and Peterson 1995). Activity in VCR alone, and possibly CCR in addition may act in a reciprocal manner, resulting in co‐contraction between agonist and antagonist muscles stabilizing the head on the trunk (Roy and Cullen 2001, 2004). Notably, unity between head and trunk motion occurred successively earlier in both groups in conditions when visual information was removed (VS) and when, in addition, attention was reduced (MA), suggesting underlying reflex activity at lower frequency perturbations, modulated by voluntary activity. No differences were found between groups in this frequency range, and the data suggest normal reflex response in patients. However, unity between head and trunk movements requires just enough muscle stiffness to keep the head stable on the trunk, and increased stiffness beyond this level does not have any significant influence on the movements. Thus, potentially increased voluntary or reflex induced muscle activity or co‐contraction in patients cannot be proven by the present method.

### Responses above 2 Hz

Above 2 Hz, amplitudes of the head exceeded the magnitude of rotations of the trunk (i.e., gain >1, left axis, Fig. 3) and studies agree on interpretations of this as an effect of mechanical resonance (Goldberg and Peterson 1986; Keshner and Peterson 1995; Peng et al. 1996; Forbes et al. 2013), meaning that the oscillatory response of a system is larger than the imposed perturbation. The resonance frequency of a mechanical system depends on the system's mass and viscoelastic properties. Increased muscle activity would increase the stiffness of the system and resonance would emerge at higher frequencies. Note, as mentioned under *measurement system limitations*, that our results related to the higher excitation frequencies should be regarded as highly uncertain.

## Conclusions

This study contributes with novel findings regarding the frequency‐dependent deficits in impaired control in patients with neck pain. The task consisted of keeping the head stable in space while the body was exposed to unpredictable horizontal perturbations of superimposed frequencies. In general, patients kept the head more stable relative to the trunk than relative to space in response to perturbations across conditions and frequencies. The largest differences compared to healthy controls were found at frequencies below 1 Hz and with a visual reference, suggesting that impaired motor control of the head and neck in patients depends on increased muscle stiffness and/or co‐activation referred to altered voluntary control. The same pattern was however also found in the condition without visual reference and with diversion from conscious control of head stability in space, thus contribution of upregulated reflex activity may partly or by itself also explains reduced ability to keep the head stable in space in patients. Reflex‐induced stabilization of the head on the trunk between 1 and 2 Hz was seen in both groups and considered normal. Reservations should be paid to that the nervous system has not been directly assessed and the relative contribution of reflexes to voluntary control cannot be determined. The relationship or interdependence between voluntary and reflex control mechanisms below 1 Hz still needs to be resolved by basic research.

## Conflict of Interest

All authors declare that they have no competing interests.

## Supporting information


**Table S1.** Data corresponding to the statistics and to the right axis in Figure 3 for gain according to the decimal logarithm on a linear scale.
